# A hepatoprotective *Lindera obtusiloba *extract suppresses growth and attenuates insulin like growth factor-1 receptor signaling and NF-kappaB activity in human liver cancer cell lines

**DOI:** 10.1186/1472-6882-11-39

**Published:** 2011-05-12

**Authors:** Christian Freise, Martin Ruehl, Ulrike Erben, Ulf Neumann, Daniel Seehofer, Ki Young Kim, Wolfram Trowitzsch-Kienast, Thorsten Stroh, Martin Zeitz, Rajan Somasundaram

**Affiliations:** 1 Department of Gastroenterology, Infectiology and Rheumatology, Charité - Universitätsmedizin Berlin, Campus Benjamin Franklin, Hindenburgdamm 30, 12203 Berlin, Germany; 2 Department of General, Visceral and Transplantation Surgery, Charité - Universitätsmedizin Berlin, Campus Virchow Klinikum, Augustenburger Platz 1, 13353Berlin, Germany; 3 Department of Surgery, University Hospital Aachen, Pauwelstraße 30, 52074 Aachen, Germany; 4 Faculty of Beauty Design, Human Environmental Science College, Wonkwang University, Iksan City, South Korea; 5 Department of Chemical and Pharmaceutical Engineering, Beuth Hochschule für Technik, Luxemburger Str. 10, 13353 Berlin, Germany

## Abstract

**Background:**

In traditional Chinese and Korean medicine, an aqueous extract derived from wood and bark of the Japanese spice bush *Lindera obtusiloba *(*L.obtusiloba*) is applied to treat inflammations and chronic liver diseases including hepatocellular carcinoma. We previously demonstrated anti-fibrotic effects of *L.obtusiloba *extract in hepatic stellate cells. Thus, we here consequently examine anti-neoplastic effects of *L.obtusiloba *extract on human hepatocellular carcinoma (HCC) cell lines and the signaling pathways involved.

**Methods:**

Four human HCC cell lines representing diverse stages of differentiation were treated with *L.obtusiloba *extract, standardized according to its known suppressive effects on proliferation and TGF-β-expression. Beside measurement of proliferation, invasion and apoptosis, effects on signal transduction and NF-κB-activity were determined.

**Results:**

*L.obtusiloba *extract inhibited proliferation and induced apoptosis in all HCC cell lines and provoked a reduced basal and IGF-1-induced activation of the IGF-1R signaling cascade and a reduced transcriptional NF-κB-activity, particularly in the poorly differentiated SK-Hep1 cells. Pointing to anti-angiogenic effects, *L.obtusiloba *extract attenuated the basal and IGF-1-induced expression of hypoxia inducible factor-1α, vascular endothelial growth factor, peroxisome proliferator-activated receptor-γ, cyclooxygenase-2 and inducible nitric oxide synthase.

**Conclusions:**

The traditional application of the extract is confirmed by our experimental data. Due to its potential to inhibit critical receptor tyrosine kinases involved in HCC progression via the IGF-1 signaling pathway and NF-κB, the standardized *L.obtusiloba *extract should be further analysed for its active compounds and explored as (complementary) treatment option for HCC.

## Background

Hepatocellular carcinoma (HCC) results from chronic liver disease and is the most common malignancy of the liver [1]. Chronic Hepatitis B or C leading to liver cirrhosis are major risk factors for the development of HCC [2]. Even in developing countries less than 40% of patients have a chance for cure when the tumor is diagnosed. In more advanced stages there are only reduced therapeutic options, since e.g. the use of more aggressive chemotherapeutic approaches is often limited by significant liver dysfunction/cirrhosis. Thus, the median survival in advanced HCC without therapy ranges from 4.2 to 7.9 months or even less [3,4]. Small molecules, targeting tumor angiogenesis, apoptosis or specific signal transduction pathways, have gained growing attention in cancer therapy. The multikinase inhibitor sorafenib is currently the only approved drug for the treatment of HCC, prolonging median survival of advanced HCC from 7.9 to 10.4 months [4]. But side effects and upcoming resistances reveal that monotherapies with the kinase inhibitors alone are not sufficient suggesting the need for combinatory and/or multitargeted therapies [5].

The receptor tyrosine kinase insulin like growth factor-1 receptor (IGF-1R) and its ligands, IGF-1 and IGF-2, are essential for cell growth and development [6] but also in the progression of various types of cancer, including HCC [7-10]. In addition, IGF-1R signaling protects cells from apoptosis mainly through the PI3K/Akt and Ras-Raf-MAPK pathways [11,12]. Activation of IGF-1R critically impacts HCC angiogenesis by induced expression of vascular endothelial growth factor (VEGF) and its transcription factor hypoxia inducible factor (HIF)-1α [13-15]. Inhibition of IGF-1R, e.g by monoclonal antibodies against IGF-1R, has been shown to block tumor growth *in vitro *and in a xenograft model of HCC and to sensitize cells for anti-tumor treatment, indicating that IGF-1R is a promising antineoplastic target [16-18]. A clinical trial targeting IGF-1R inhibition is currently ongoing in patients with advanced solid tumors. Preliminary data suggest evidence of clinical activity and good tolerance [19].

IGF-1R signaling via the PI-3K/AKT-axis also impacts the nuclear factor-kappaB (NF-κB), which is not only considered a key factor in inflammation but also regulates angiogenesis and as a major characteristic mediates inhibition of apoptosis [20]. NF-κB is spontaneously activated in HCC cells [21,22] and induces expression of cyclooxygenase-2 (COX-2) or inducible nitric oxide synthase (iNOS) which support cell survival and might contribute to the resistance against exogenously induced tumor cell apoptosis [23,24].

Traditionally, Oriental medicine makes use of compositions from or mixtures of different plants to prevent or to treat cancer and liver diseases [25,26]. Novel multitargeted therapeutics including natural compounds such as epigallocatechin-3-gallate from green tea have gained growing attention [27].

In traditional Chinese and Korean medicine preparations from *Lindera obtusiloba *(*L.obtusiloba*) comprise a good physiological compatibility and are applied to treat inflammations and to improve blood circulation [28]. Especially in Korean medicine an extract of *L.obtusiloba *is used for a long time for the treatment of chronic liver diseases which includes treatment of HCC the endstage of chronic liver disease (personal communication, Prof. Ki Young Kim, Wonkwang University, Korea).

Bioactive components from the leaves of *L.obtusiloba *described so far exert cytotoxicity against tumors as shown with human cancer cell lines from lung (A549), ovarian cancer (SK-OV-3), skin (SK-MEL-2), the central nervous system (XF498) or colon (HCT15) with half-maximum inhibitory concentration (IC_50_) values ranging from 3-20 μg/ml of the respective compounds [29,30]. We previously found an aqueous extract from wood and bark of *L.obtusiloba *to suppress profibrotic stimuli, exerting anti-oxidative activity, reduction of the expression of pro-fibrotic marker proteins and inhibition of matrix-metalloproteinases in hepatic stellate cells [31]. In addition, this extract displayed anti-inflammatory and anti-adipogenic activity in 3T3-L1 preadipocytes [32].

However, experimental data from in vitro or in vivo studies on anti-neoplastic effects of *L.obtusiloba *extracts in human HCC as complication of chronic liver disease were not reported so far. We here used well established HCC cell lines that represent diverse stages of differentiation and different degrees of invasiveness to examine direct anti-neoplastic effects of *L.obtusiloba *extract, standardized to its antiproliferative and anti-fibrotic effects, on tumor cells and to get insights into signaling pathways involved. With a clear focus on aspects affecting angiogenesis and tumor cell invasion, we aimed to understand mechanisms of action of *L.obtusiloba *extract.

## Methods

### Materials and reagents

Tissue culture plates and polystyrene microtiter for ELISA as well as for fluorimetric analysis were from Nunc (Roskilde, Denmark) and Dynex (Chantilly, VA), respectively. If not stated otherwise, all reagents were purchased from Merck (Darmstadt, Germany) or Sigma-Aldrich (Deisenhofen, Germany) and were of the highest purity available. Cell culture media and solutions were purchased from Invitrogen (Karlsruhe, Germany) or Biochrom (Berlin, Germany).

### Preparation and standardization of *L.obtusiloba *extract

Freeze-dried extracts of *L.obtusiloba *were obtained as described previously [31]. To obtain stock solutions, 10 mg powder was redissolved in 10 ml sterile phosphate-buffered saline (PBS) at 60°C for 30 min. Aliquots were stored at -20°C. Freshly prepared working solutions of *L.obtusiloba *extract were routinely standardized according to their anti-fibrotic and anti-inflammatory activity as previously described [31,32]. Briefly, 100 μg/ml *L.obtusiloba *extract had to reduce proliferation of 3T3-L1 preadipoctyes by 45% and to suppress the autocrine stimulation of TGF-β expression of hepatic stellate cells by 50% before to be used in the assays with HCC cells.

### Cell culture

The human HCC cell lines HepG2 (ATCC HV-8062), Hep3B (ATCC HV-8064), Huh-7 (JCRB 0403; Tokio, Japan) and SK-Hep1 (ATCC HTB-52) cells (Fuchs et al., 2008) were cultured in a humidified atmosphere at 37°C and 5% CO_2_. Standard culture medium consisted of DMEM with 862 mg/l L-alanyl-L-glutamine, 4.5 g/l glucose, 50 μg/ml streptomycin, 50 units/ml penicillin, 50 μg/ml L-ascorbic acid, supplemented with 10% heat-inactivated fetal bovine serum (FBS). Cell layers were detached with 0.05% trypsin/0.02% EDTA solution. Cell morphology in culture was directly examined by inverse phase contrast microscopy (Zeiss, Oberkochen, Germany).

### HCC cell proliferation

HCC cells (5 × 10^3^) were seeded into 96-well tissue culture plates in 100 μl standard culture medium. After 24 h, cells were cell cycle synchronized in 100 μl culture medium containing 0.2% FBS for additional 24 h. Cultures were treated with up to 200 μg/ml *L.obtusiloba *extract as indicated for 20 h. Proliferation was determined by adding 0.5 μCi/well [^3^H]-thymidine (GE Healthcare, Munich, Germany) for 4 h. Cells were fixed with 10% trichloro acetic acid and the DNA was solubilized with 200 mM NaOH, neutralized with an equal volume of 800 mM HCl and transferred to glass filter pads. Radioactive decay was monitored by liquid β-scintillation counting within 1 min (LKB Wallac Turku, Finland).

### Cell invasion assays

50 μl of 3 mg/ml Matrigel™ (BD Biosciences, Heidelberg, Germany) diluted in ice cold, serum free DMEM were used to coat the upper compartments of 24-well transwell inserts (BD Biosciences; pore size 8 μm) for 16 h at 37°C. 2 × 10^5 ^cells diluted in 300 μl serum free medium were seeded into the upper compartments and *L.obtusiloba *extract was added at a final concentration of 100 μg/ml. DMEM containing 10% FBS as stimulating agent was added to the lower compartment and the plates were incubated for up to 24 h at 37°C in a humidified atmosphere with 5% CO_2_. Cells that remained in the upper compartment were gently removed with a cotton swab. The inserts were then washed with PBS and invaded cells on the lower surface of the insert were fixed for 20 min with 2% glutaraldehyde in PBS and stained using 0.1% crystal violet in water. The stained cells on each insert were visualized by light microscopy and manually counted in three independent spots per insert.

### Apoptosis by caspase 3/7 activity

Apoptosis was quantified fluorimetrically from caspase-3/7 activity. In brief, 2 × 10^5 ^HCC cells in standard culture medium were seeded into 24-well tissue culture plates. Confluent cell layers were thoroughly washed with DMEM and subsequently incubated with culture medium containing 0.2% FBS for 24 h. Cells were then treated for another 24 h in the presence of 100 μg/ml L.obtusiloba extract or 100 nM staurosporine and 0.2% FBS. Apoptosis was determined using the SensoLyte™ Homogenous AFC Caspase-3/7 Assay Kit (AnaSpec, San Jose, CA) according to the manufactures instructions. Briefly, cells were lysed in 200 μl lysis buffer for 1 h at 4°C. The clear supernatant obtained after centrifugation at 2,500 × g for 30 min was stored at -80°C until measurement. Caspase 3/7-mediated conversion of the substrate N-acetyl-Asp-Glu-Val-Asp-7 amino-4 trifluoromethyl coumarin was monitored fluorometrically using a Spectramax Gemini EM microplate reader (λex: 380 nm, λem: 500 nm; Molecular Devices, Sunnyvale, CA).

### Western-blot

HCC cells cultured in 6-well tissue culture plates with 125 ng/ml human recombinant IGF-1 (Biomol, Hamburg, Germany), 100 μg/ml *L.obtusiloba *extract and a combination of both for 48 h were rinsed with ice-cold PBS and lysed with a lysis-buffer containing 50 mM Tris-HCl pH 7.4, 2.25 M urea, 1.4% sodium dodecyl sulfate, 100 mM dithiothreitol, 2 mM NaVO_3_, 5 mM NaF, and per 10 ml buffer one tablet of Complete Mini Protease Inhibitor cocktail (Roche, Penzberg, Germany). Aliquots of 333 μl lysate were transferred to 0.5 ml reaction tubes and frozen at 80°C. Protein content was determined using the Nano Orange Protein Assay Kit (Molecular Devices) according to the manufactures instructions. From each cell lysate, 25 μg protein per lane were separated by SDS-PAGE and transferred to nitrocellulose membranes (Bio-Rad, Munich, Germany) using a tank blot apparatus (Hoefer, Holliston, MA). Membranes blocked for 1 h with 5% skim milk powder in a buffer containing 10 mM Tris, 154 mM NaCl, 0.1% Tween 20 were incubated over night at 4°C with the following specific primary antibodies with the dilution given: Akt (1:1,250), COX-2 (1:1,000), Erk1/2 (1:1,000), iNOS (1:1,500), pAkt (1:1,250), pErk1/2 (1:1,250), Stat3 (1:1,250; Cell Signaling, Beverly, MA), β-Actin (1:10,000), HIF-1α (1:2,000; Novus Biologicals, Littleton, CO, USA), IGF-1R (1:1,250), pIGF-1R (1:1,250; Imgenex, San Diego, CA) and PPARγ (1:2,000), pStat3 (1:1,250), VEGF (1:800; Santa Cruz, Santa Cruz, CA). After washing, membranes were incubated for 1 h with rabbit or mouse immunoglobulin G-specific horseradish peroxidase-labeled secondary antibodies (1:2,500; Dako, Hamburg, Germany). Bands were detected by enhanced chemiluminescence (GE Healthcare) using the Luminescent Image Analyser LAS-4000 (Fujifilm, Düsseldorf, Germany). Band intensities were quantified using Image J and normalized to the β-actin loading control.

### Transient transfection of HCC cells

Transfection of the cells was performed using the electroporation method and a NF-κB-luciferase reporter plasmid as described by Stroh et al. [33,34]. Detached cells (2 × 10^5^) were resuspended in 100 μl electroporation buffer containing 90 mM phosphate buffer pH 7.2, 10 mM MgCl_2_, and 50 mM glucose before 4 μg of the NF-κB-luciferase reporter plasmid pNF-κB-TA-Luc (Clontech, Mountain View, CA) were added. In an electroporation cuvette with a gap of 2 mm (Biozym, Hessisch Oldendorf, Germany), cells were subjected to single square pulses of 400 V for 400 μs (HepG2, Hep3B and Huh-7) or 600 V for 400 μs (SK-Hep1), allowed to rest for 1 min, and transferred into prewarmed standard culture medium. A total of 1 × 10^5 ^transfected cells in 1 ml culture medium were seeded into a 24-well plate. Cell viability as determined by Calcein AM staining [32] was about 85% in conjunction with a cell transfection efficacy of ~75%.

### Assessment of NF-κB activation by luciferase assay

Twenty hours after transfection with the NF-κB-luciferase reporter plasmid [33] cells were treated with 10 μg/ml recombinant human TNFα (Peprotech, Hamburg, Germany), 100 μg/ml *L.obtusiloba *extract, a combination of both and 15 nM of the NF-κB inhibitor 17-Dimethylamino-ethylamino-17-demethoxygeldanamycin (17-DMAG, InvivoGen, San Diego, CA). Cells were incubated for 24 h, washed twice with PBS, and lysed in 80 μl of reporter lysis buffer (Promega, Mannheim, Germany). Protein concentrations were determined using the Nano Orange Protein Assay Kit. Samples (20 μl) were transferred into a white 96 well plate before 60 μl of luciferase substrate were added and mixed for 5 s. Luciferase activity was measured for 0.5 s using a Mithras LB 940 luminescence reader (Berthold Technologies, Bad Wildbad, Germany). NF-κB activity was estimated as relative luminescence units (RLU) corresponding to equal protein amounts.

### Statistical Analysis

One way ANOVA/Tukey Tests were performed using SigmaStat for Windows (version 2.03; Systat, San Jose, CA). P < 0.05 was considered significantly different.

## Results

### *L.obtusiloba *extract reduces proliferation, induces apoptosis and blocks invasion of HCC cells

**Figure 1:**
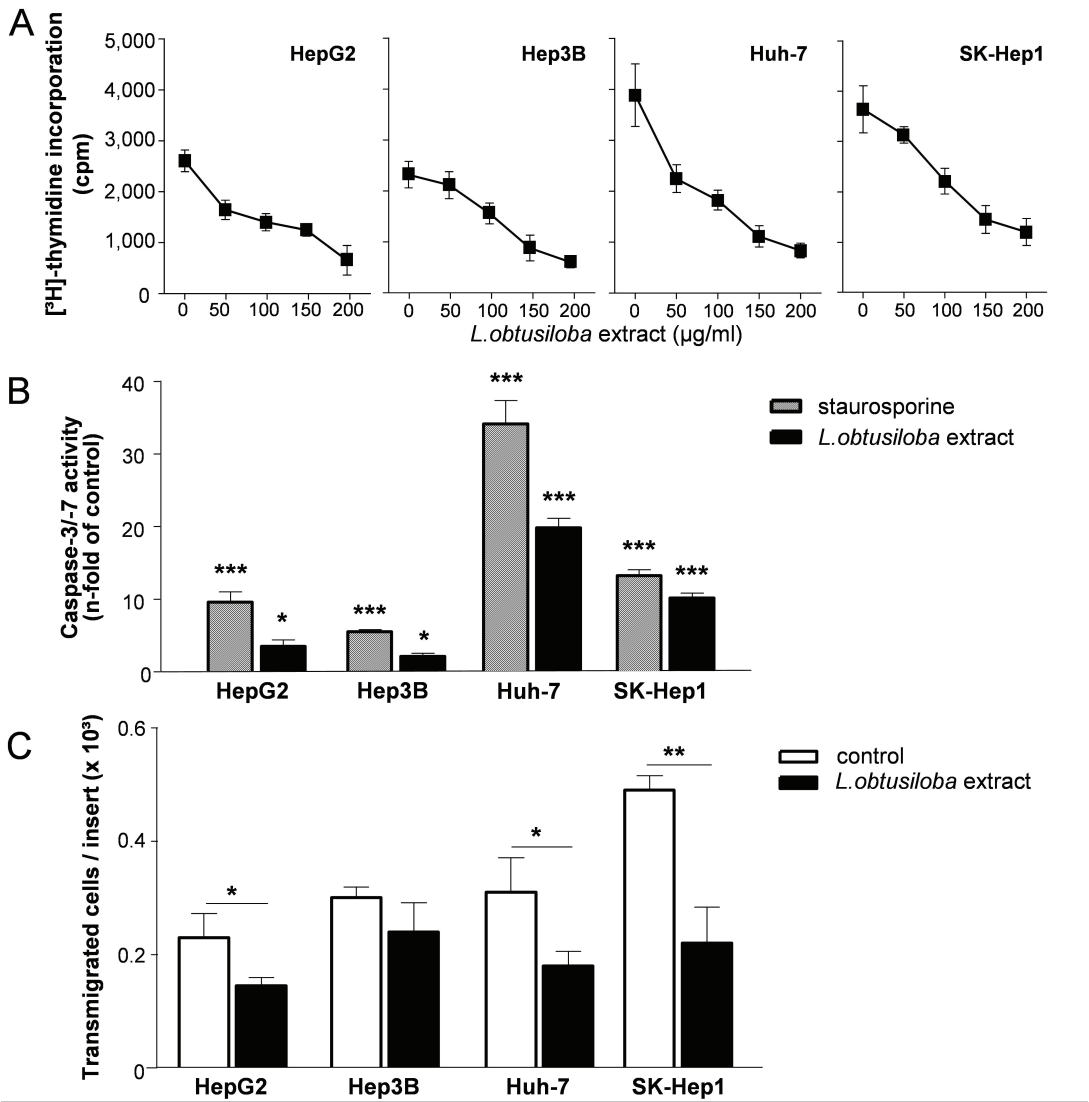
**Proliferation, apoptosis and invasion of HCC cell lines treated with *L.obtusiloba *extract**. (A) Cell cycle synchronized HepG2, Hep3B, Huh 7 and SK-Hep1 cells were treated with up to 200 μg/ml *L.obtusiloba *extract for 24 h. Cultures without *L.obtusiloba *extract served as controls. Proliferation was determined by [^3^H]-thymidine incorporation within the last 4 h of the culture. Mean values ± SD of three parallel measurements. (B) HCC cells were incubated with 100 μg/ml *L.obtusiloba *extract for 24 h. Cultures without additives or with 100 nM staurosporine served as negative or positive controls, respectively. Enzymatic activities of caspase-3/7 were determined from cell lysates by fluorogenic substrate conversion. Shown are the mean values ± SD of four parallel measurements. (C) HCC cells were allowed to invade membranes coated with basement collagen in the absence or presence of 100 μg/ml *L.obtusiloba *extract. After 24 h, transmigrated cells were stained with crystal violet and numbers were counted. Shown are the mean values ± SD of three independent experiments with four parallel measurements.*P < 0.05, **P < 0.01, ***P < 0.001.

Effects of *L.obtusiloba *extract on the proliferation of human HCC cells were tested in cell-cycle synchronized cell lines. To define effective dose ranges, HCC cells in culture were treated with up to 200 μg/ml *L.obtusiloba *extract (Figure 1A). The range of concentration of *L.obtusiloba *extract and the experimental protocols were adapted from preceding studies dealing with the extract [31,32].

*L.obtusiloba *extract reduced the proliferation of all four human HCC cell lines in a dose-dependent manner. The IC_50 _values for the inhibition of the de novo DNA synthesis were approximately 100 μg/ml *L.obtusiloba *extract for all HCC cell lines. This concentration was used in all subsequent experiments. Induction of apoptosis due to exposure of cells with *L.obtusiloba *extract was determined by the enzymatic activity of pro-apoptotic caspase-3/-7 (Figure 1B). As shown for the apoptosis inducer and kinase inhibitor staurosporine used as control, all cell lines were highly susceptible to induction of apoptosis by *L.obtusiloba *extract as shown by 2.2- to 20-fold enhanced caspase activity. In the differentiated HCC cell lines HepG2, Hep3B and Huh-7, this effect of *L.obtusiloba *extract did not exceed 60% of the effect of 100 nM staurosporine. In contrast, *L.obtusiloba *extract provoked a caspase activity that corresponded to ~80% of apoptosis induced by staurosporine in the poorly differentiated SK-Hep1 cells (P < 0.001). Since their migratory potential mainly defines their aggressiveness, 100 mg/ml *L.obtusiloba *extract was applied to HCC cells in matrigel invasion assays. Again, while *L.obtusiloba *extract only slightly attenuated the invasion of HepG2, Huh-7 (P < 0.05) and Hep3B cells through a reconstituted basement membrane, it led to a stronger reduction of invasion in SK-Hep1 cells by 55% (P < 0.01) (Figure 1C). As for direct effects of *L.obtusiloba *extract on tumor cells, it diminished the invasive potential of HCC cell lines and was most effective on cells displaying a highly aggressive phenotype.

### *L.obtusiloba *extract reduces basal and IGF-1-induced protein expression of VEGF and its transcription factor HIF-1α

HCC represents a highly vascularized tumor entity and the tumor cells contribute to that process by production of proteins regulating angiogenesis. Thus, we next investigated whether *L.obtusiloba *extract impacts the expression of VEGF and HIF-1α in HCC cell lines. Linking Huh-7 to SK-Hep1 cells, stimulation with exogenous IGF-1 enhanced basal expression of VEGF by 1.4- or 3.3-fold, while in HepG2 and Hep3B no effects of IGF-1 were observed (Table 1). *L.obtusiloba *extract alone reduced VEGF expression in all four cell lines but strongest in Huh-7 cells. In combination with IGF-1, *L.obtusiloba *extract did not affect the IGF-1-induced VEGF expression in HepG2 cells, but in Hep3B, Huh-7 and SK-Hep1. The IGF-1-induced enhancement of HIF 1α expression was most prominent in differentiated HepG2 cells (3.6-fold) and intermediate in Hep3B (1.5-fold) and SK-Hep1 cells (1.3-fold). In Huh-7 cells no significant IGF-1-mediated effects on HIF 1α expression were observed. Similar to VEGF, *L.obtusiloba *extract distinctly reduced basal and IGF-1-induced HIF-1α expression in each of the HCC cell lines to comparable individual levels that were independent of the presence of IGF-1. These findings on VEGF and HIF-1α pointed to a strong anti-angiogenic potential of *L.obtusiloba *extract. Consequently, we studied the impact of *L.obtusiloba *extract on the expression of other proteins crucial in neo-angiogenesis.

**Table 1:** Expression of VEGF and HIF-1α in human HCC cell lines

	VEGF expression			HIF-1α expression		
		
	IGF-1	*L.obtusiloba *extract	IGF-1 and *L.obtusiloba *extract	IGF-1	*L.obtusiloba *extract	IGF-1 and *L.obtusiloba *extract
HepG2	1.02 ± 0.03	0.75 ± 0.10*	0.93 ± 0.06	3.58 ± 0.26*	0.72 ± 0.07*	0.82 ± 0.11^#^
Hep3B	0.82 ± 0.18	0.67 ± 0.09*	0.54 ± 0.10*,^#^	1.52 ± 0.21*	0.62 ± 0.11*	0.63 ± 0.07*,^#^
Huh-7	1.38 ± 0.05*	0.28 ± 0.10*	0.47 ± 0.08*,^#^	0.89 ± 0.12	0.14 ± 0.04*	0.05 ± 0.03*,^#^
SK-Hep1	3.28 ± 0.24*	0.93 ± 0.10	0.83 ± 0.09^#^	1.28 ± 0.13*	0.67 ± 0.09*	0.68 ± 0.12*,^#^

Whole cell lysates from cells treated with 100 μg/ml *L.obtusiloba *extract, 125 ng/ml human IGF-1 or a combination of both for 48 h and from untreated cells as control were analyzed by western-blot specific for VEGF and HIF-1α. β-Actin was stained for equal loading control and specific band intensities were normalized to β-actin. VEGF and HIF-1α protein expression levels were calculated in relation to the respective untreated cells. Mean values ± SD from three independent experiments. *P < 0.05 compared to the untreated control, ^# ^P < 0.05 compared to IGF-1-treated cells.

### *L.obtusiloba *extract decreases the protein expression of PPARγ, COX-2 and iNOS

The expression of the nuclear transcription factor PPARγ and its target genes COX-2 and iNOS are implicated in hepatocarcinogenesis and in the formation of enhanced microvessel density in HCC tissues. Effects of *L.obtusiloba *extract on the expression of PPARγ, COX-2 and iNOS were examined at protein level (Table 2). The expression of PPARγ in all four HCC cell lines was enhanced after stimulation with IGF-1. *L.obtusiloba *extract reduced both, basal and IGF-1-induced PPARγ expression with the same pattern as HIF-1α (Table 1). COX-2 was not detected in HepG2 and Huh-7 cells (Table 2). On the other hand, Hep3B and SK-Hep1 showed a high IGF-1-induced expression of COX-2 by 2.3- and 3.2-fold, respectively and with *L.obtusiloba *extract a reduction of both, the basal and the IGF-1-induced COX-2 expression. Hep3B and Huh 7 cells showed no expression of iNOS. In HepG2 and SK-Hep1 cells the basal expression of iNOS was enhanced by IGF-1 by 1.2- and 1.9-fold, respectively. *L.obtusiloba *extract reduced the basal and the IGF-1-induced iNOS expression of both cell lines by ~80%.

**Table 2:** Expression of PPARγ, COX-2 and iNOS in human HCC cell lines

	PPARγ expression			COX-2 expression			iNOS expression		
			
	IGF-1	*L.obtusiloba *extract	IGF-1 and *L.obtusiloba *extract	IGF-1	*L.obtusiloba *extract	IGF-1 and *L.obtusiloba *extract	IGF-1	*L.obtusiloba *extract	IGF-1 and *L.obtusiloba *extract
HepG2	4.31 ± 0.51*	0.76 ± 0.14	1.15 ± 0.09^#^	n.d.	n.d.	n.d.	1.17 ± 0.07	0.21 ± 0.14*	0.21 ± 0.09*;^#^
Hep3B	1.33 ± 0.12*	0.84 ± 0.09	0.81 ± 0.05^#^	2.28 ± 0.19*	0.77 ± 0.08*	1.09 ± 0.04*;^#^	n.d.	n.d.	n.d.
Huh-7	1.17 ± 0.05	0.21 ± 0.10*	0.31 ± 0.12*;^#^	n.d.	n.d.	n.d.	n.d.	n.d.	n.d.
SK-Hep1	1.43 ± 0.11*	0.75 ± 0.09*	0.89 ± 0.07^#^	3.21 ± 0.34*	0.80 ± 0.07*	0.82 ± 0.09^#^	1.87 ± 0.12*	0.27 ± 0.12*	0.35 ± 0.06*;^#^

Cell lysates of HCC cell lines as described for Table 1, were subjected to specific western-blots for PPARγ, COX 2 and iNOS and for β actin as equal loading control. Densitometry of specific band intensity was normalized to β-actin expression. Mean values ± SD from three independent experiments. PPARγ, COX-2 and iNOS protein expression levels were calculated in relation to the respective untreated cells. Mean values ± SD from three independent experiments. *P < 0.05 compared to the untreated control, ^# ^P < 0.05 compared to IGF-1-treated cells, n.d. - not detected

Taken together and complementing the results from the preceding experiments, these data suggest direct effects of *L.obtusiloba *extract on the angiogenic program of HCC cells via decreased expression of PPARγ and its target genes COX-2 and iNOS thus contributing to dampened growth and motility of HCC cells.

### *L.obtusiloba *extract blocks expression of VEGF and HIF-1α via attenuated activation of IGF 1R downstream targets

**Figure 2:**
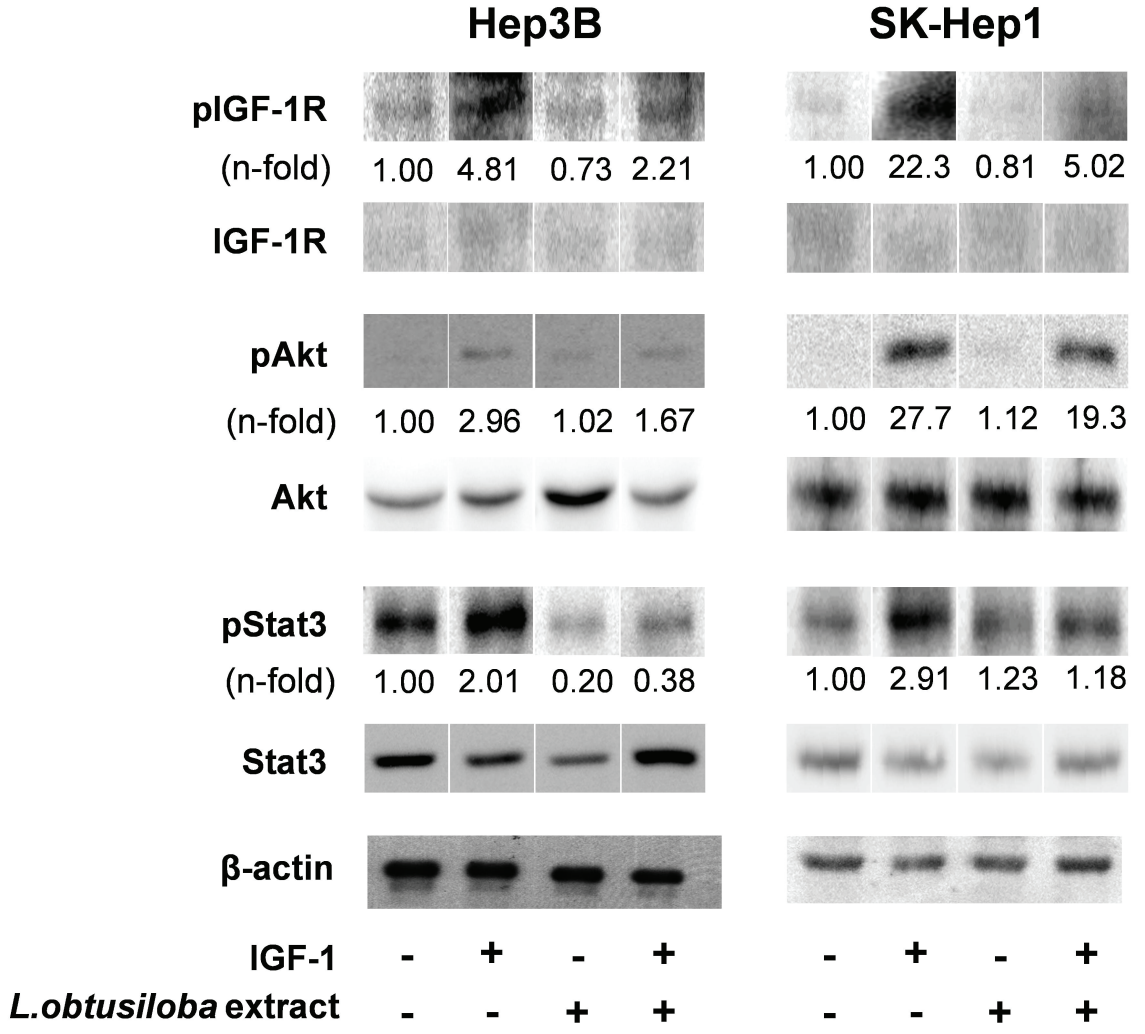
**Effects of *L.obtusiloba *extract on the basal and IGF-1-induced IGF-1R signal transduction**. As described for Table 2, HCC cells treated for 24 h with 100 μg/ml *L.obtusiloba *extract, 125 ng/ml IGF-1, a combination of both or untreated cells were lysed. Whole cell lysates were analyzed by western-blot with antibodies specific for phosphorylated and normal IGF-1R and its downstream targets Akt and Stat3. Bands were visualized by chemiluminescence. Blots are representative for three independent experiments. Quantification of the protein-activation (n-fold to control values) were taken from table 3.

The IGF-1/IGF-1R axis plays an important role in angiogenesis and therefore the development of HCC. To investigate signaling pathways involved, western-blots specific for (p)IGF-1R and the activation states of its target proteins were focused on Hep3B as one out of the three less invasive HCC cells (compare Figure 1C) and the more aggressive SK-Hep1 cells. In both cell lines 100 μg/ml exogenous IGF-1 increased the phosphorylation state of the IGF-1R (Figure 2, Table 3). This IGF-1-mediated activation of the IGF-1R was strongly reduced in the presence of *L.obtusiloba *extract; by half in the Hep3B and to about a quarter in SK-Hep1 cells. As for the downstream signaling molecules Akt, Stat3 and Erk, *L.obtusiloba *extract did not alter basal phosphorylation. IGF-1 induced phosphorylation of Akt, Stat3 and Erk were tested in both cell lines. Increased pAkt levels that were at least partially abrogated by *L.obtusiloba *extract were found to be the most prominent effect. Treatment with *L.obtusiloba *extract in combination with IGF-1 markedly decreased the levels of pAkt, pStat3 and pErk in Hep3B and SK-Hep1 cells.

**Table 3:** Effects of *L.obtusiloba *extract on basal and IGF-1-induced signal transduction via IGF-1R

	Hep3B			SK-Hep1		
		
	IGF-1	L.obtusiloba extract	IGF-1 and L.obtusiloba extract	IGF-1	L.obtusiloba extract	IGF-1 and L.obtusiloba extract
pIGF-1R	4.81 ± 0.40*	0.73 ± 0.08*	2.21 ± 0.31*; #	22.3 ± 1.98*	0.81 ± 0.07	5.02 ± 0.60*; #
pAkt	2.96 ± 0.25*	1.02 ± 0.10	1.67 ± 0.19*; #	27.7 ± 2.84*	1.12 ± 0.09	19.3 ± 1.45*; #
pStat3	2.01 ± 0.18*	0.20 ± 0.07*	0.38 ± 0.21*; #	2.91 ± 0.22*	1.23 ± 0.20	1.18 ± 0.19#
pErk2	1.32 ± 0.14*	0.13 ± 0.03*	0.57 ± 0.07*; #	2.81 ± 0.32*	1.01 ± 0.10	1.82 ± 0.17*; #

**Figure 3:**
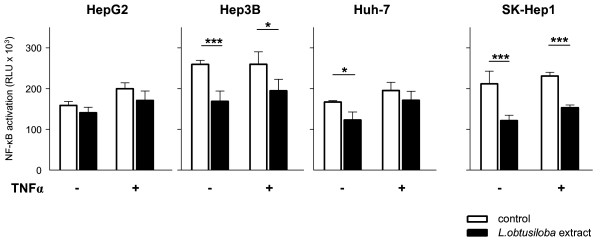
**NF-κB activity of HCC cell lines in the presence of *L.obtusiloba *extract**. HCC cell lines were transfected with a NF-κB/luciferase reporter plasmid and allowed to adhere for 20 h before being treated with 10 μg/ml TNFα, 100 μg/ml *L.obtusiloba *extract or a combination of both. Untreated cells served as negative controls. After additional 24 h, cells were lysed and luciferase activity was determined. Shown are the mean values ± SD of four parallel measurements. *P < 0.05, ***P < 0.001.

HCC cells were treated with 100 μg/ml *L.obtusiloba *extract, 125 ng/ml IGF 1, a combination of both or remained untreated for 24 h. Phosphorylation of IGF-1R and its downstream targets Akt, Stat3 and Erk were studied in whole cell lysates by specific western-blot analysis as shown in representative blots in Fig. 3. Activation of the proteins was determined from densitometric assessment in comparison to total expression levels of the respective non-phosphorylated protein. Mean values ± SD in relation to untreated cells from three independent experiments. *P < 0.05 compared to the untreated control, ^# ^P < 0.05 compared to IGF-1-treated cells.

These findings that *L.obtusiloba *extract decreased the basal phosphorylation of Akt, Stat3 and Erk in Hep3B cells as well as in poorly differentiated SK-Hep1 cells as a result of reduced stimulatory effects of IGF-1 on its receptor explains the inhibition of growth and motility and the induction of apoptosis in HCC cells.

### *L.obtusiloba *extract decreases transcriptional activity of NF-κB

NF-κB is a key regulator of crucial pro-inflammatory cytokines during carcinogenesis and promotes cell survival and angiogenesis. Since *L.obtusiloba *extract induces apoptosis (Figure 1B) and displays anti-inflammatory activity [32], we assessed whether the extract decreases the activity of NF-κB in HCC cells (Figure 3). All four HCC cell lines transfected for transient constitutive expression of NF-κB exhibited high levels of basal NF-κB transcriptional activity of about 160 260 RLU. This activity was not significantly increased by addition of TNFα. In all cell lines, treatment of transfected cells with the specific NF-κB-inhibitor 17-DMAG reduced the activity to <10% of the basal level thus approving the function of the experimental system (data not shown). Except for HepG2 cells, *L.obtusiloba *extract attenuated the transcriptional activity of NF-κB to 75% (P < 0.05) of the basal level in Huh-7 and to ~65% (P < 0.001) in Hep3B cells while in the poorly differentiated SK-Hep1 cells the high basal transcriptional activity of NF-κB was reduced to 50% (P < 0.001). These results at the level of regulation clearly strengthen our conclusion that *L.obtusiloba *extract directly impairs the survival and the angiogenic program in HCC cells.

## Discussion

In the present study with human HCC cell lines we provide evidence that a well standardized aqueous extract from wood and bark of *L.obtusiloba *exerts direct and non-direct anti-neoplastic effects via attenuated IGF-1R- and NF-κB-signaling.

Initially, we examined the effects of a standardized active extract of *L.obtusiloba *on the proliferation of well characterized human HCC cell lines with poorly differentiated SK-Hep1 considered more aggressive than the other three used. *L.obtusiloba *extract blocked the growth of the HCC cells in a dose dependent manner with a physiologically relevant IC_50 _of ~100 μg/ml (Figure 1A) [31,32]. In addition, *L.obtusiloba *extract inhibited tumor cell invasion (Figure 1C). Here, SK-Hep1 cells rather than the well differentiated HepG2, Hep3B and Huh-7 cells were more sensitive to *L.obtusiloba *extract. Thus, in conjunction with the induction of apoptosis in all four cell lines (Figure 1B), *L.obtusiloba *extract exerts three primary prerequisites for the treatment of cancer [35,36].

Aberrant growth and apoptosis regulation in carcinogenesis is mediated by growth factor receptors such as IGF-1R which therefore represents an attractive therapeutic target [8,37] and all of the four cell lines investigated are known to express the IGF-1R [38]. Since HCC is characterized by strong neo-angiogenesis [39] with VEGF as its main mediator we investigated the upstream IGF-1/IGF-1R signal transduction and the expression of VEGF via induction of HIF-1α [13]. *L.obtusiloba *extract blocked the basal and IGF-1-induced protein expression of HIF-1α and VEGF accompanied by decreased phosphorylation of Akt, Stat3 and Erk. (Figure 2, Tables 1, 2, 3). Since a forced activation of Akt, Stat3 and Erk was shown to protect from apoptosis and to induce VEGF expression [40,41], our results suggest that a decreased activation of the IGF-1/IGF-1R-axis due to *L.obtusiloba *extract treatment contributes to its apoptosis-inducing effects and might be a reason for the reduced expression of VEGF and HIF-1α in HCC cells treated with *L.obtusiloba *extract [11,12]. These findings are in accordance with studies using extracts from green tea describing a decreased expression of VEGF and HIF-1α accompanied by a block of PI3K/Akt-signaling in HCC cells [42].

IGF-1R signaling also impacts the expression of the transcription factor PPARγ which in turn modulates the expression of other angiogenesis-regulating proteins like COX-2 and iNOS. The implication of PPARγ in carcinogenesis is still debated. Some data show anti-tumor effects of PPARγ ligands. However, these effects could also be independent of PPARγ activation and in addition the usage of PPARγ antagonists also exerts anticancer effects [43]. In contrast to PPARγ, several studies clearly show a positive correlation between the expression of COX-2 and iNOS and HCC progression, e.g. indicated as enhanced microvessel density in HCC [44]. While COX-2 impacts growth and progression of HCC and its inhibition suppressed HCC-associated angiogenesis *in vitro *and *in vivo *[45], iNOS is a key enzyme in generating nitric oxide, thus modulating tumorigenesis by regulating tumor cell proliferation, survival and migration, as well as angiogenesis, drug resistance and DNA repair [5,46].

In line with previous reports [47,48], *L.obtusiloba *extract reduced the expression of COX-2 and iNOS (Table 2). Notably, poorly differentiated SK-Hep1 cells were susceptible to IGF-1 and inhibition of IGF-1 by *L.obtusiloba *extract. A similar result was obtained for the expression of PPARγ (Table 2). We therefore conclude that downregulation of COX 2 and iNOS by *L.obtusiloba *extract is mediated by diminished expression of PPARγ.

Beside PPARγ, IGF-R-signaling, through different upstream pathways, could trigger the activation of the transcription factor NF-κB [49] which likewise regulates COX-2 and iNOS and plays a role in viral hepatitis, chronic liver disease including fibrosis and cirrhosis and in HCC [24,50] and is spontaneously activated in HCC cells [22]. Inhibition of NF-κB reduced proliferation and invasion as well as expression of VEGF in HCC cells and sensitized the cells to sorafenib induced cell death [51].

As shown in Figure 3, *L.obtusiloba *extract markedly reduced the transcriptional activity of NF-κB in Hep3B, Huh-7 and SK-Hep1 cells and to a lesser extent in HepG2 cells. Thus, downregulation of COX-2 and iNOS by *L.obtusiloba *extract is mediated by diminished expression of PPARγ and due to a reduced transcriptional activity of NF-κB. Since NF-κB activity supports cell survival or entails anti-apoptotic effects [23,24,49], the inhibition of NF-κB by *L.obtusiloba *extract might contribute to the apoptosis inducing effects of the extract in the cancer cells (Figure 1B).

In summary, our findings *in vitro *strongly suggest *L.obtusiloba *extract as a specific compound to suppress tumor cell growth and migration and to induce apoptosis in aggressive, poorly differentiated human tumor cells via attenuation of NF-κB transcriptional activity and IGF-1R signaling. Further, the expression of key proteins in regulation of angiogenesis was reduced due to *L.obtusiloba *extract treatment. Due to its good physiological compatibility, in Korea *L.obtusiloba *extract is traditionally applied in humans to treat chronic inflammatory diseases of the liver [28]. Thus, our *in vitro *results are in line with and add more scientific strength to the traditional use of *L.obtusiloba *extract in treatment for chronic liver disease including HCC.

Regarding biologically active compounds in the extract several studies describe the isolation and structural characterization of drugs from *Lindera obtusiloba *[29,30,52]. In this line, preliminary data of us suggest that lignans such as sesamin or episesamin might contribute to the anti-fibrotic and anti-tumor effects of *L.obtusiloba *extract (not shown).

Complemental to the anti-fibrogenic, anti-inflammatory and anti-adipogenic efficacy of *L.obtusiloba *extract [31,32], our results suggest the use of an inflammation-associated tumor model of HCC to assess all aspects of the anti-tumor effects of *L.obtusiloba *extract *in vivo*.

## Conclusions

Due to its potential to inhibit critical receptor tyrosine kinases involved in HCC progression via the IGF-1 signaling pathway and NF-κB, we conclude that *L.obtusiloba *extract or its active compounds represent a useful tool in a rational complementary approach e.g. with sorafenib for treatment of HCC or as cancer preventive agents.

## Competing interests

The authors declare that they have no competing interests.

## Authors' contributions

CF participated in the design and coordination of the study, carried out the analyses and wrote the manuscript. MR and UE helped to draft the manuscript. UN and DS provided the HCC cell lines and helped to draft the manuscript. KK helped to prepare the *L.obtusiloba *extract and helped to draft the manuscript. WTK helped to prepare the *L.obtusiloba *extract and participated in the design of the study. TS designed the cell transfection experiments. MZ helped to draft the manuscript. RS participated in the data interpretation and manuscript preparation. All authors read and approved the final manuscript.

## Pre-publication history

The pre-publication history for this paper can be accessed here:

http://www.biomedcentral.com/1472-6882/11/39/prepub
